# Decisive or impulsive? Re-examining Africa’s lockdown response to COVID-19

**DOI:** 10.1186/s41182-022-00414-7

**Published:** 2022-03-08

**Authors:** Aishat Jumoke Alaran, Abubakar Olaitan Badmos, Oumnia Bouaddi, Yusuff Adebayo Adebisi, Kenechukwu Ben-Umeh, Umarfarouq Idris, Don Eliseo Lucero-Prisno

**Affiliations:** 1 Global Health Focus, Rwanda, Rwanda; 2 National Primary Healthcare Development Agency, Abuja, Nigeria; 3 University Mohammad V, Rabat, Morocco; 4 Department of Pharmacotherapy, University of Utah, Salt Lake City, USA; 5 National Agency for Food and Drug Administration and Control, Abuja, Nigeria; 6 Department of Global Health and Development, London School of Hygiene and Tropical Medicine, London, UK

**Keywords:** Novel coronavirus disease, COVID-19, Pandemic, Africa, Lockdown, Response

## Abstract

Due to the high transmission rate and mortality index of the current coronavirus pandemic, many settings in Africa instituted lockdowns to reduce its rate of spread and avert exponential growth rate. At the early stage, this measure seemed to heighten awareness of the virus and subsequently minimized exponential growth of cases. However, these lockdowns have had great consequences on the weak health systems and frail economy in place in many African countries. In this paper, we examine the impact of lockdown measures in these countries and provides key recommendations in dealing with present and future pandemics.

## To the editor

Amidst the huge global burden of pandemic proportions caused by the novel coronavirus disease, different measures including those that are prudent and productive and those that are abrupt and inconsequential have been taken to curtail its ravaging effects. African countries have not been spared of the negative consequences of the novel coronavirus disease: since the first index case reported in Egypt on the 14th of February 2020, more than eight million cases and 220,658 deaths have been documented in the continent (as at 11th November, 2021) [1]. A disease with aggressive rate of spread and severity, the causative virus could not be contained within Wuhan, where it originated in December, 2019 [2]. It soon spread, first, to Thailand, then to France and the United States of America in January 2020, and then to Egypt in February 2020 [3–6]. Deeply concerned by this alarming transmissibility and its high mortality index, and having established evidence of human-to-human transmission, the World Health Organisation (WHO) identified 13 priority African countries (Algeria, Angola, Cote d’Ivoire, Democratic Republic of Congo, Ethiopia, Ghana, Kenya, Mauritius, Nigeria, South Africa, Tanzania, Uganda, Zambia) on 5th February, 2020. These countries either have direct links or a high volume of travel to China [7]. Early measures taken in these priority countries to avert the spread of the coronavirus disease to these countries included enhanced surveillance at airports and screening of passengers with history of travel to China. In spite of these measures, the index case was imported to the continent through Egypt [4].

The following weeks after the report of the index case saw a cataclysm of events unfolding—over 40 cases were reported in nine different African countries including Algeria, Cameroon, Egypt, Morocco, Nigeria, Senegal, South Africa, Togo and Tunisia [4]. To respond to this new reality, on 3rd February, 2020, the African Union constituted the African Task Force for Coronavirus which was charged with implementation of the continental strategy devised to avert exponential epidemic growth trajectories. The task force harnessed and leveraged existing continental expertise to collaborate with Centre for Disease Control (CDC), WHO and African Union (AU) member states and partners [8]. This synergy, after workshops and webinars, concentrated efforts on implementing further control measures by adopting lockdown protocols which had already been popular in other regions of the world.

Consequently, many countries in the continent enforced stringent lockdown protocols, usually with punitive measures for violators, at a time when there was no clear definition of what lockdown actually or fully entails, and when only little amount of knowledge about the COVID-19 disease was obtainable [9]. At the early stage, this measure seemed to heighten awareness of the virus and subsequently minimized exponential growth of cases. However, the aftermaths of these lockdowns were of extreme consequences on the health systems, the economy, and the population [10, 11]. For instance, there were cases of police aggression in form of physical and sexual harassments reported in Nigeria [12]. Similar cases of violence and humiliation were reported in South Africa, Kenya and Zimbabwe [13]. In view of the recurrent break-out of newer variants of the disease, and the propensity of many African countries to revisit the lockdown blueprint, we take an insight on the downfalls of lockdown protocols in the African context, unpack the many consequences it has yielded, and derive lessons that can be learned when developing strategic approaches for adequate preparedness for the ongoing and next pandemics.

## Good lockdowns, bad lockdowns: why context matters

Conceptually speaking, lockdowns are an emergency, albeit transient, response imposed indiscriminately by the government to mandate people to stay indoor in the event of an outbreak of a disease or social unrest [9]. The high transmissibility of COVID-19 and the fact the mode of transmission was largely unknown meant that standard communicable disease control including active case detection, contact tracing, selective isolation and quarantine may be insufficient to bring transmission under control [9, 14]. Therefore, countries began to implement lockdowns to curtail indiscriminate spread of the virus.

The first lockdown response to COVID-19 was implemented in Wuhan, China, at the beginning of the pandemic. This measure saw a significantly reduced transmission and was dubbed ‘an unprecedented public health response’ by the WHO [15]. In developed countries that have been the epicenters of the pandemic ebb, and where lockdowns are observed, there are pre-existing strong health systems, buoyant economy and abundance of resources including food and shelter. Furthermore, specific measures are intermittently deployed to mitigate the adverse effects of lockdown, including provision of relief funds to individuals, supporting small and medium-scale businesses, dispensing personal protective equipment to essential workers and (re)allocation of budget to combat the pandemic [16].

In majority of African countries, however, the health and economic situation vis-à-vis the availability of resources is far different compared to the high-income countries. There is huge level of poverty and financial insecurity; social protection services are largely weak; civil unrest and war are worrisomely persistent; healthcare systems are severely underdeveloped; the trust in government is low; and there are recurrent cases of epidemic diseases, such as Ebola and Lassa fever, and endemic diseases, such as malaria, HIV and tuberculosis—diseases that hitherto constitute negative impacts to the African healthcare systems [17–19]. It is, therefore, manifest that measures such as lockdown that have the potentials to incapacitate access to health, trigger economic recession, disrupt supply systems, aggravate social tension, and lead to severe erosion of human rights and civil liberties should be approached with discretion and utmost consideration of on-ground situations [9, 20].

Despite the overwhelming presence of evidence of the catastrophic impacts unplanned lockdown can have, most countries close down schools and businesses, and prohibited public and private gatherings to varying degrees. South Africa instituted a countrywide curfew by the end of March, 2020 [21]. Uganda mandated home confinement and curfew between hours of 19:00 and 6:30 [22]. In Nigeria, a stay-at-home order was issued in three states and while most of the other states followed suits by imposing curfews with varying leeway [23]. Ghana ordered a lockdown in its two largest metropolitan areas for a 3-week period only [24]. In Zimbabwe, formal sector businesses were closed down between 08:00 and 15:00[25]. Sudan and Sierra Leone implemented similar country-wide curfews [9]. These lockdown measures have variable lengths, peculiarities and durations. However, one commonality that can be inferred from these lockdowns is a lack of co-ordination and sense of direction, as evidenced by inconsistent bans and unnecessary flexibilities. It reflects unplanned decision made without systems put in place to monitor its effectiveness on disease control and impacts on the populace. The exacerbated aftermaths are discussed in the next section.

## Negative impacts of lockdown measures in Africa

Available evidences show that lockdown measures observed in most African countries are largely ineffectual due to the ripples of inconsistencies in adherence and the overall guidelines of the protocols, and therefore, they have widely insignificant impact on growth of COVID-19 cases [9]. It can be suggested that the low number of cases recorded is due, in part, to inadequate detection as a result of low testing rate. In a striking contrast, number of cases seen in developed countries is heavily dependent on the number of tests done [26]. It is in fact plausible to surmise that in the dense, semi-formal settlement of many settings in Africa, where high number of individuals live together in single households, the coronavirus has a higher tendency to spread among them [27]. In the absence of any other evidence, we can extrapolate that lockdowns in these African settings have negative impact on COVID-19 control against which it is originally implemented.

The impact on health system is pronounced, foremostly, by the obvious difficulty of access to healthcare. Patients are either forced away from hospitals out of fear of contracting the disease or are directly prevented from seeking healthcare by curfews and its accompanying harsh enforcement. There was 10–36% increase in malaria-related deaths during this period, while reports of its complications, such as deafness and other neurological deficits spiked [28]. The sudden interruption of anti-retroviral and anti-tuberculous medications constituted another negative impact of the lockdown—a recipe for deterioration of illness, increase of deaths from the diseases and development of drug resistance [17]. Another backlash of lockdowns is exacerbations of the delays causing maternal mortality. The three important delays—including delay to seek help; delay to reach help; and delay to get help—are evidently accentuated by lockdown measures [29]. Consequently, this threatens the huge achievement made in reducing maternal mortality rate over the years and impedes further progress. In the same vein, routine immunization delivery was immensely interrupted during the lockdowns [30, 31]. Numerous cases of defaulters were reported, signaling a looming health crisis of resurgence of preventable diseases which have been latent in the population for a long period.

New data on income streams under lockdowns in sub-Saharan African countries show that 9.1% of the population have immediately fallen into extreme poverty as a result of the pandemic, with about 65% of this increase resulting from lockdown [32]. Food supply chains were exposed, with a significant proportion of individuals falling into starvation. The world bank estimated that millions of people will experience abject poverty as the lockdown policies, closure of borders and restrictions on transportations resulted in the disruption of the food supply chains and the slowdown of the economic activities [33, 34]. Most governments revealed a drop in national Gross Domestic Product (GDP). For example, Nigeria estimated a 38% drop in GDP during the first 5-week lockdown [35]. Debts rose due to reduced revenues by taxes and increased spending on fiscal and monetary policies. Jobs were lost in droves—studies have estimated that between 2.2 and 2.8 million jobs were lost in South Africa from February to April 2020 [36]. The World Bank predicted in April 2020 that the economy across the continent could contract by as much as 5.1% due to a decline in output growth among the region’s key trading partners, a fall in commodity prices, reduced tourism activity (e.g., in Sudan, Tanzania and Uganda) as well as lower levels of foreign direct investment and remittances [37]. On all fronts, it is clear that the economic effects of lockdowns in Africa are catastrophic.

Furthermore, the livelihoods of citizens are severely impaired by the lockdown. There was a huge and disproportionate imposition of restrictions on personal freedoms and civil liberties, as well as the suspension of democratic procedures and safeguards. This illegal erosion of citizens’ rights emboldened the government to exert unfair power on them, implying negative political consequences. There were numerous occasions of rising violence and conflicts between armed officials and citizens perceived to violate lockdown rules; for instance, just 2 weeks after the enforcement of lockdown in Nigeria, 18 people were killed by security forces in the country [38]. In addition, lockdowns enabled increased rates of domestic violence among intimate partners—with girls and women usually victims—which increased social inequalities. Closing down of schools grossly impaired learning and facilitated eventual dropping outs and overall disruption of curriculums [9, 39]. Finally, many studies have noted the implication of the pandemic and the subsequent lockdowns enforced in many countries on the mental health of the populace as stress and uncertainty resulting from job loss of jobs and livelihoods and limited access to social support structures due to restrictions could be overwhelming for many and could, therefore, leads to mental health deterioration [9, 36].

## Recommendations

While Africa has been largely spared the impact that has thrown other countries into complete turmoil, the indirect impact of COVID-19 on its population is unprecedented due to the already existing disproportionate burden of disease, deplorable infrastructures, constrained resources and fragile economy [40]. The root of mitigating the overall impact of the pandemic in Africa—as opposed to just the direct impact—lies in not making decisions against the backdrop of priorities at home. The adoption of home-grown, country-specific solutions that are based on grassroots experience and that take into account the diversity across and within the continent, the unique social arrangements and the lack of resources is critical to balancing the impact of the pandemic with other health priorities [41].

Some key recommendations should be considered by policymakers and public health officials to increase pandemic preparedness. In low-resource settings, the building of new infrastructure overnight, and making mass purchases of beds, equipment and diagnostic kits is a daunting challenge; however, Africa has built up expertise in confronting diseases and managing outbreaks over the years. In this context, African countries should leverage the resources at hand, such as polio infrastructure and taskforce, to increase surveillance and engage in active case finding to contain outbreaks effectively. It is equally important to involve community health workers in the surveillance, case reporting and containment of clusters of cases.

Moreover, the role of coordinated global support and south-to-south collaboration in strengthening African health systems cannot be overemphasized as is evidenced by the hard-won gains in improved health outcomes made thanks to the global initiatives against Human Immunodeficiency Virus (HIV), malaria, tuberculosis and Ebola as well as Global Polio Eradication Initiative and partners. It is also worthy of note that youth account for almost 60% of the total population in Africa and should thus be endorsed as key stakeholders in pandemic preparedness and response as well as in combating misinformation, including vaccine hesitancy, by disseminating information in their local communities and on the internet [42].

Reduced access to outpatient care is much more devastating in Africa than in high-income countries due the simultaneous increase in the prevalence of non-communicable diseases alongside the existing high disease burden attributable to infectious diseases. It is, therefore, imperative that access to essential services and out-patient care is preserved [43]. In addition, the scaling up of epidemic intelligence systems and the building of robust surveillance systems using informational and technological systems is needed to confront infectious disease threats of local and international origins. Sub-Saharan African governments also need to make substantial investments in capacity building and research infrastructure and support local institutions that conduct genomic research as they could become focal points for research into future epidemic or pandemics [44].

Furthermore, health interventions in Africa are unfortunately not spared from political interference, which thereby makes rolling out, testing, tracing, and care across Africa challenging. Strengthening political leadership in health governance is thus urgently required to create a conducive environment to incentivize local production of drugs, vaccines and diagnostic tools, promote health research and increase public trust in public health institutions.

Finally, efforts must be concentrated on strengthening the economic condition in the continent. Not surprisingly, more than 70% of people living below the global poverty line are found in Africa [45]. Economic policies targeted at alleviating poverty, creating employment opportunities and encouraging even distribution of resources must, therefore, be implemented to create an environment that is ready and economically strong to withstand the negative impacts of disease outbreaks.

## Conclusions

Most countries including those in Africa have significantly eased lockdowns. However, in the face of continuing evolution of the pandemic, and despite the discovery of vaccines, there is a growing possibility for reestablishment of lockdowns in majority of African countries. What these countries must consider, however, are evidence-based consequences of the erstwhile, unpremeditated lockdown measure which was characterized by profound, multiple health and socioeconomic implications. This paper highlights the collateral damage incurred by the lockdown and suggests the need for rigorous and contextual consideration of the African situation when planning for measures against outbreaks. Appropriate policies, backed with well-informed research, must be prioritized; robust and scientifically sound techniques should be adopted; and adequate reparations must be urgently made to remedy the burden instigated by the lockdown.

## Data Availability

Data sharing not applicable to this article as no data sets were generated or analysed in the current study.

## Abbreviations

AU: African Union
CDC: Centre for Disease Control
GDP: Gross Domestic Product
HIV: Human immunodeficiency virus
WHO: World Health Organisation
